# Differential effects and mechanisms of local anesthetics on esophageal carcinoma cell migration, growth, survival and chemosensitivity

**DOI:** 10.1186/s12871-020-01039-1

**Published:** 2020-05-25

**Authors:** Guanhua Zhu, Ling Zhang, Jiapeng Dan, Qiong Zhu

**Affiliations:** 1 Department of Anesthesiology, Jingzhou Central Hospital, Jingzhou, Hubei Province China; 2 Department of Anesthesiology, The First Affiliated Hospital of Yangtze University, Hangkong Road 8, Jingzhou, 434020 Hubei Province China

**Keywords:** Local anesthetics, Esophageal carcinoma, Mitochondria, Rac1

## Abstract

**Background:**

Retrospective analysis and pre-clinical studies suggest that local anesthetics have anti-tumoral effects. However, the association between cancer recurrence and the use of local anesthesia is inconclusive and most reports are based on single local anesthetic results.

**Methods:**

The biological effects (growth, migration and survival) of four common local anesthetics on esophageal carcinoma cells were compared. Biochemical assays on molecules involved in cell migration and proliferation were analyzed.

**Results:**

Ropivacaine and bupivacaine significantly inhibited esophageal carcinoma cell migration, at clinically relevant micromolar concentrations. Mepivacaine and lidocaine showed less potent cell migration inhibition than ropivacaine or bupivacaine. All four local anesthetics inhibited cell proliferation. Of note, the effective concentration of anti-proliferative activities requires higher doses. At millimolar concentrations of these local anesthetics, cell apoptosis was moderately affected. Drug combination analysis demonstrated that two of four local anesthetics augmented chemotherapeutic drugs in inhibiting migration. However, all four local anesthetics significantly augmented chemotherapeutic drugs in inhibiting growth and inducing apoptosis. The anti-growth and anti-survival effects of four local anesthetics were attributed to mitochondrial dysfunction and oxidative damage. The anti-migratory effect of local anesthetics is likely through decreasing Rac1 activity.

**Conclusions:**

Our work demonstrates the differential effects and proposes the mechanisms of local anesthetics on esophageal carcinoma cell migration, growth, survival and chemosensitivity.

## Background

Esophageal cancer is the sixth leading cause of cancer-related mortality and the eighth most common cancer worldwide. Despite advances in diagnostics and therapeutics, the five year survival rate in esophageal carcinoma remains poor [1]. Surgery remains the main curative option for advance local esophageal cancer to improve patient survival. However, local-regional disease recurrences and distant organ metastases are found in a significant proportion of patients after surgical procedures [2]. Studies suggest that anesthetics used in the perioperative period can potentially influence cancer recurrence [3, 4]. Certain local anaesthesia reduce cancer recurrence in a number of retrospective studies. However, these studies have small sizes and are subjected to selection bias [4–7].

Mepivacaine, bupivacaine, ropivacaine and lidocaine are amide-linked local anesthetics and often used during the perioperative period in cancer patients [8, 9]. Amide local anesthetics act on nerve cells through blocking voltage-gate sodium-channels, resulting in the decreased rate of depolarization and repolarization of excitable nerve cell membrane [10]. Substantial preclinical studies suggest that local anesthetics have direct inhibitory effects on the biological activities of cancer cells, including cell proliferation, migration, invasion and survival [11–15]. The mechanisms of the action of the local anesthetics in cancer cells are via targeting multiple signaling or related molecules, and furthermore are sodium-channel-independent [16, 17]. Ropivacaine has been recently reported to inhibit esophageal cancer cell migration via prenylation-dependent inhibition of Rac1/JNK/paxillin [16]. However, systematic evaluation and comparison of these commonly used local anesthetics in esophageal carcinoma biological functions are lacking.

In this work, we investigated the effects of four local anaesthetics on esophageal carcinoma cell growth, survival and migration, as well as their combinatory effects with chemotherapeutic drugs. We show that the four local anesthetics 1) have differential inhibitory effects on esophageal carcinoma cells in aspects of effective doses, cell lines and cellular activities; 2) acts differently with chemotherapeutic drugs; 3) acts on esophageal carcinoma cells via varying mechanisms.

## Methods

### Cell culture

Esophageal carcinoma cell lines OE19 and SK-GT-4 (Sigma, USA) were cultured in 75 ml flasks at 37 °C with 5% CO_2_, using Dulbecco’s Modified Eagle Medium supplemented with penicillin at 100/ml, streptomycin at 100 μg/ml and10% heat-inactivated fetal bovine serum (FBS) (Invitrogen, USA). The cell lines used in our study are validated with short tandem repeat (STR) profile analysis.

### Drugs

Local anesthetics including lidocaine, mepivacaine, bupivacaine and ropivacaine were obtained from the Department of Pharmacy, Jingzhou Central Hospital. 5-Fluorouracil (5-FU) and paclitaxel were purchased from Sigma, USA.

### Measurement of proliferation

Cells were seeded onto a 96-well plate with up to 50% confluency. Different agents were added to the culture plate the next day. These included different local anaesthetics at varying concentrations, single chemotherapeutic drug, or their combinations. After 72 h incubation, cell proliferation activity was determined by the BrdU Cell Proliferation Assay Kit (Cell Signaling, USA).

### Measurement of apoptosis

Cells were seeded onto a 6-well plate with up to 50% confluency. Different agents were added to the culture plate the next day. These included different local anaesthetics at varying concentrations, single chemotherapeutic drug, or their combinations. After 72 h incubation, cells were harvested using trypsin for apoptosis analysis using Annexin V/7-AAD kit. The Annexin V-positive cells were determined by analysing cells on Beckman Coulter FC500 Flow Cytometer (Beckman Coulter, USA).

### Boyden chamber migration assay

Migration assay was performed using the same method as described in our previous studies [13]. Briefly, pre-treated cells suspended in 2% FBS medium were seeded onto the cell culture inserts and 10% FBS medium as attractant was placed onto the lower chamber. Migration was captured for 8 h. The migrated cells were stained with 0.4% crystal violet and counted under microscope. Cell number of five random fields were quantified.

### Measurement of oxygen consumption rate (OCR)

OCR was measured using a Seahorse XF24 extracellular flux analyser (Seahorse Bioscience, USA) as described in our previous studies [13]. Briefly, after 24 h drug treatment on XF24 tissue culture plates, cells were equilibrated to the un-buffered medium in a CO_2_-free incubator. OCR was then measured on Seahorse Analyzer at basal condition.

### ELISA assays

Cells were incubated with drug for 24 h and were then harvested and homogenized using a standard protocol. Cell lysates were adjusted to the same concentration using PBS. Cellular RhoA and Rac1 activity were assessed using total cell lysates and were determined using RhoA G-LISA Activation Assay Kit and Rac1 G-LISA Activation Assay Kit (Cytoskeleton, Inc. USA). Active RhoA and Rac1 level were measured on absorbance at 490 nm. The level of 8-hydroxydeoxyguanosine (8-OHdG) which is a ubiquitous marker of oxidative DNA damage was measured on absorbance at 450 nm as per protocol and reagents provided by OxiSelect™ Oxidative DNA Damage ELISA Kit (Cell Biolabs Inc., USA).

### Measurement of reactive oxygen species (ROS)

Cells were incubated with drug for 24 h and were then harvested and homogenized using a standard protocol. ROS level was measured by using DCFDA/H2DCFDA - Cellular ROS Assay Kit (Abcam, USA) as per manufacture’s protocol. The absorbance was measured on a fluorescence plate reader at ex/em of 495/525 nm.

### Statistical analyses

The data are expressed as mean and standard deviation (SD). For comparison between groups of two categorical variables, these were analysed by the Student’s t test. Across multiple groups, one-way analysis of variance (ANOVA) with post-hoc Tukey was performed. A *p*-value < 0.05 was considered statistically significant.

## Results

### The differential effects of local anesthetics on esophageal carcinoma cell migration

**Fig. 1:**
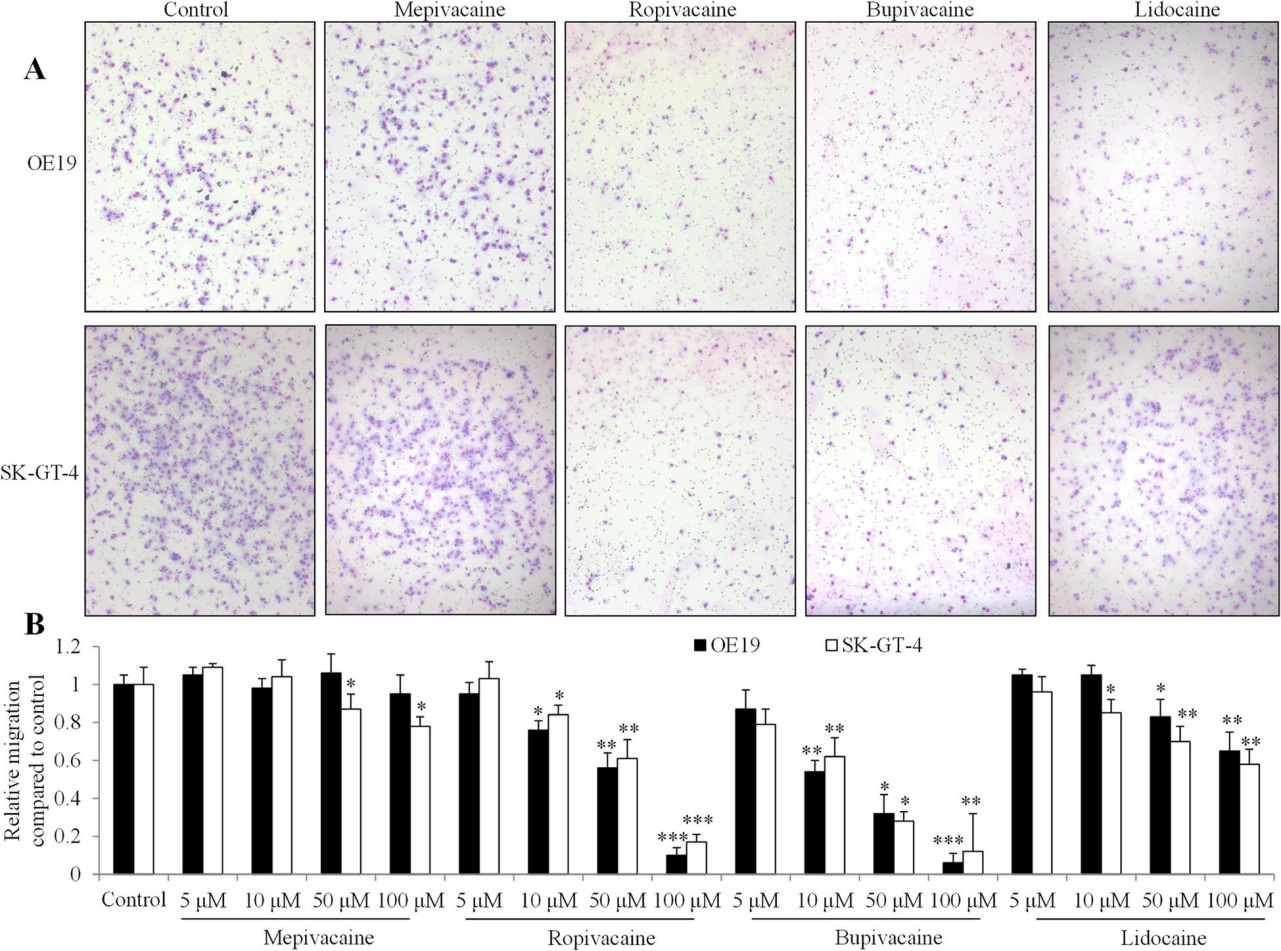
The differential effects of local anesthetics on esophageal carcinoma cell migration. **a** Representative photos of cell migration assay. OE19 and SK-GT-4 were treated with control or four local anesthetics at 100 μM: mepivacaine, bupivacaine, ropivacaine and lidocaine. **b** Quantification of cell migration using the Image J software shows the differential degree of inhibition on esophageal carcinoma cells among four local anesthetics at concentration range from 5 to 100 μM. Cell number of five random fields were quantified. *P < 0.05, **P < 0.01, ***P < 0.001, compared to control. All experiments were independently performed at least three times

To establish the effects of local anesthetics on esophageal carcinoma metastasis, we conducted the Boyden Chamber migration assay using OE19 and SK-GT-4 cells in the presence of local anesthetics: mepivacaine, bupivacaine, lidocaine and ropivacaine. OE-19 was derived from an adenocarcinoma of a gastric/esophageal junction and SK-GT-4 was derived from a well-differentiated adenocarcinoma of the distal esophagus. Both are used as representatives of esophageal adenocarcinoma. We exposed esophageal carcinoma cells to local anesthetics at micromolar concentrations. Ropivacaine and bupivacaine were observed to significantly inhibit migration of both OE19 and SK-GT-4 cells in a dose-dependent manner, starting from 10 μM (Fig. 1). Of note, both ropivacaine and bupivacaine at 100 μM resulted in ~ 80% inhibition of migration. In contrast, lidocaine significantly inhibited migration of both OE19 and SK-GT-4 cells starting from 50 μM and lidocaine at 100 μM. These resulted in ~ 30% inhibition. Interestingly, mepivacaine inhibited migration of SK-GT-4 but not OE19 cells. Mepivacaine at 100 μM resulted in only ~ 20% inhibition. These results demonstrated that ropivacaine and bupivacaine were much more potent than lidocaine and mepivacaine in inhibiting migration of esophageal carcinoma cells. In addition, OE19 and SK-GT-4 cells responded to mepivacaine for cell migration inhibition in a different manner.

### The differential effects of local anesthetics on esophageal carcinoma cell growth and survival

**Fig. 2:**
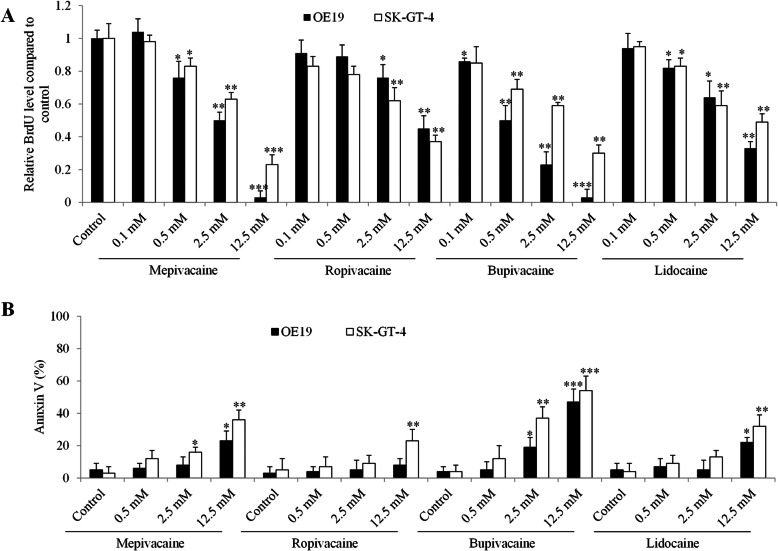
The differential effects of local anesthetics on esophageal carcinoma cell growth and survival. The differential degree of growth inhibition (**a**) and apoptosis induction (**b**) among four local anesthetics at concentration range from 0.1 to 12.5 mM in esophageal carcinoma cell. *P < 0.05, **P < 0.01, ***P < 0.001, compared to control. All experiments were independently performed at least three times

We next employed the BrdU incorporation assay and Annexin V labeling method to assess the proliferation and apoptosis of esophageal carcinoma cells exposed to local anesthetics. Based on our previous findings on the varying effective concentrations of bupivacaine on gastric cancer cell migration, growth and survival [13], we treated cells with local anesthetics at millimolar concentrations. We found that all tested local anesthetics significantly inhibited both OE19 and SK-GT-4 cell proliferation in a dose-dependent manner (Fig. 2a). In addition, OE19 was more sensitive to mepivacaine and bupivacaine than SK-GT-4 cells whereas OE19 and SK-GT-4 responded similarly to lidocaine and ropivacaine. Of note, mepivacaine and bupivacaine at 12.5 mM achieved ~ 100% growth inhibition whereas lidocaine and ropivacaine at 12.5 mM achieved ~ 50% growth inhibition in OE19 cells.

In contrast to growth inhibition, we observed a slight to modest apoptosis induction by all tested local anesthetics. Mepivacaine at 12.5 mM induced ~ 20% and ~ 40% apoptosis in OE19 and SK-GT-4 cells (Fig. 2b). Ropivacaine at 12.5 mM induced ~ 20% apoptosis in SK-GT-4 cells and did not affect OE19 survival. Bupivacaine at 12.5 mM induced ~ 40% and ~ 50% apoptosis in OE19 and SK-GT-4 cells. Lidocaine at 12.5 mM induced ~ 20% and ~ 30% apoptosis in OE19 and SK-GT-4 cells. We noted that ropivacaine at 2.5 mM and bupivacaine at 0.5 mM significantly decreased proliferation while sparing apoptosis (Fig. 2). Cell cycle analysis indicated that ropivacaine at 2.5 mM and bupivacaine at 0.5 mM increased G2/M percentage, suggesting that the cell cycle was arrested in G2/M phase by these two local anaesthetics (Fig. S1). Altogether, three of four tested local anesthetics up to 2.5 mM did not induce apoptosis in esophageal carcinoma cells.

### The differential combinatory effects of local anaesthetics with chemotherapy drugs on esophageal carcinoma cell migration, growth and survival

**Fig. 3:**
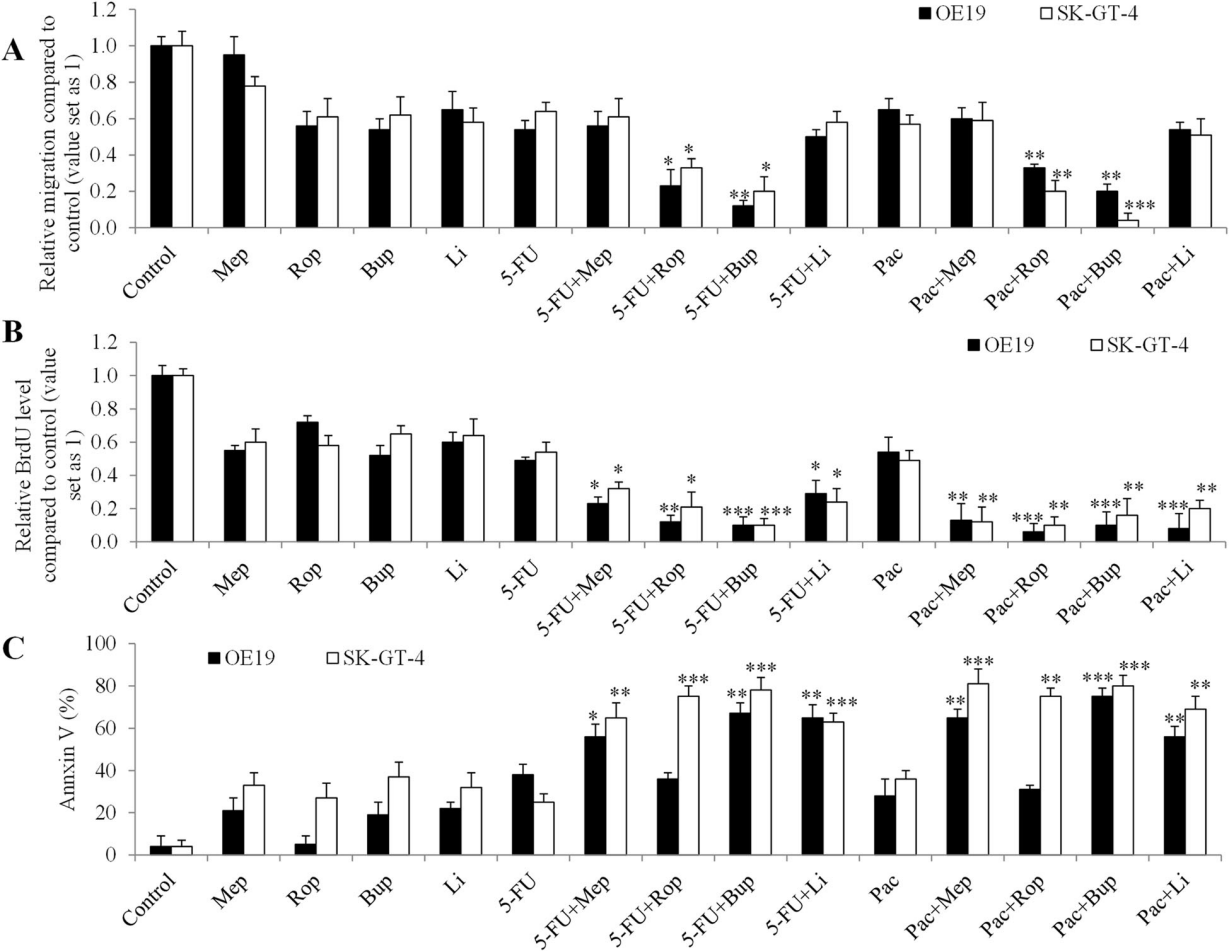
The differential combinatory effects of local anesthetics on esophageal carcinoma cell migration, growth and survival. **a** Bupivacaine (Bup) and ropivacaine (Rop) but not mepivacaine (Mep) or lidocaine (Li) significantly augment the effects of 5-FU and paclitaxel in decreasing esophageal carcinoma cell migration. 100 μM of mepivacaine and lidocaine, 50 μM of ropivacaine and 10 μM of bupivacaine were used. All four local anesthetics significantly augment the effects of 5-FU and paclitaxel in decreasing esophageal carcinoma cell in inhibiting proliferation (**b**) and inducing apoptosis (**c**). 2.5 mM of mepivacaine, ropivacaine and lidocaine, and 0.5 mM of bupivacaine were used in proliferation assay. 12.5 mM of mepivacaine, ropivacaine and lidocaine, and 2.5 mM of bupivacaine were used in apoptosis assay. 5-FU at 100 nM and paclitaxel at 50 nM were used for the combination studies in migration, proliferation and apoptosis assays. *P < 0.05, **P < 0.01, ***P < 0.001, compared to paclitaxel or 5-FU alone. All experiments were independently performed at least three times

To investigate whether local anaesthetics influence the efficacy of chemotherapy in esophageal carcinoma cells, we performed combination studies using local anaesthetics and commonly used chemotherapeutic drugs: 5-FU and paclitaxel. The concentration of anaesthetics and chemotherapeutic agent in earlier analyses that led to around 50% inhibition on cell proliferation, survival and migration as a single drug alone was chosen for the combination studies. We found that the combination of ropivacaine or bupivacaine with 5-FU or paclitaxel were significantly more effective in inhibiting migration of esophageal carcinoma cells than 5-FU or paclitaxel alone (Fig. 3a). However, combination of lidocaine or mepivacaine with 5-FU or paclitaxel inhibited cell migration in a similar manner as the chemotherapeutic drugs alone. These suggest that some but not all local anesthetics displays synergism with chemotherapeutic agents in inhibiting esophageal carcinoma cell migration.

We further observed that all tested local anaesthetics significantly augmented the anti-proliferative and pro-apoptotic effects of 5-FU or paclitaxel in esophageal carcinoma cells (Fig. 3b and c). It was noted that the combination of local anaesthetics with chemotherapeutic drugs resulted in ~ 90% growth inhibition and ~ 80% apoptosis induction in SK-GT-4 cells, suggesting the remarkably enhanced effects between local anesthetics and chemotherapy. In addition, we observed that ropivacaine failed to augment the pro-apoptotic effect of 5-FU and paclitaxel in OE19 cells.

### The differential mechanisms of local anaesthetics’ action in esophageal carcinoma cells

**Fig. 4:**
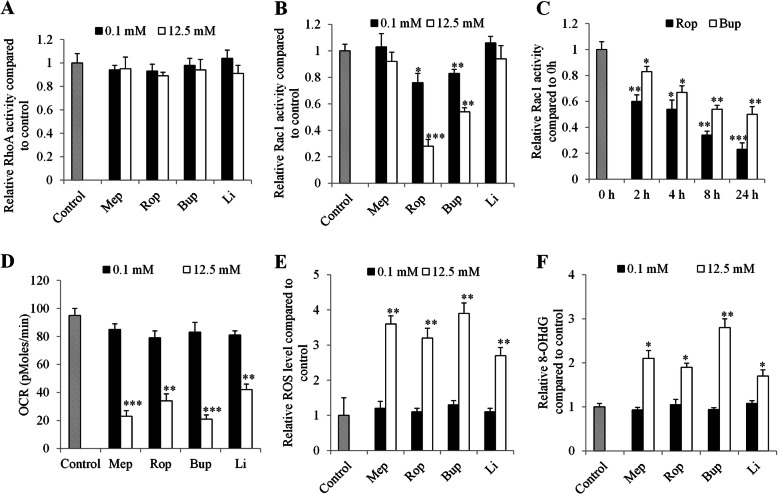
The differential mechanisms of local anesthetics’ action in esophageal carcinoma cells. **a** Local anesthetics up to 12.5 mM do not affect RhoA activity in SK-GT-4 cells. Ropivacaine and bupivacaine but not lidocaine or mepivacaine significantly decreases Rac1 activity (**b**) in SK-GT-4 cells. **c** Ropivacaine and bupivacaine decreased Rac1 activity in a time-dependent manner. Local anesthetics at 12.5 mM but not 0.1 mM significantly decreases OCR level (**d**) and increases ROS level (**e**) and 8-OHdG level (**f**) in SK-GT-4 cells. *P < 0.05, **P < 0.01, ***P < 0.001, compared to control. All experiments were independently performed at least three times

To investigate the mechanisms of local anesthetics’ action in esophageal carcinoma cells, we examined the activities of essential molecules involved in cell migration, such as RhoA and Rac1 [18, 19]. We found that all tested local anesthetics did not affect RhoA activity in SK-GT-4 cells (Fig. 4a). Ropivacaine and bupivacaine but not mepivacaine or lidocaine at both 0.1 mM and 12.5 mM significantly decreased Rac1 activities (Fig. 4b). Time course analysis indicated that ropivacaine and bupivacaine decreased Rac1 activity as early as 2-h drug treatment (Fig. 4c).

Local anesthetics have been shown to interact with mitochondria and affect mitochondrial respiratory chain with compromised mitochondrial functions [20, 21]. We found that bupivacaine, mepivacaine, ropivacaine and lidocaine at 2.5 mM and 12.5 mM but not 0.1 mM significantly decreased OCR level, increased intercellular ROS and 8-OHdG levels (Fig. 4d-f and Fig. S2 to S4). Of note, all local anesthetics at concentrations that decreased proliferation significantly inhibited mitochondrial respiration, increased ROS and 8-OHdG levels (Fig. S2 to S4), suggesting that proliferation inhibition by local anesthetics might be due to their ability in inducing mitochondrial dysfunction, oxidative stress and damage in esophageal carcinoma cells.

## Discussion

Although earlier small retrospective clinical trials suggest the beneficial roles of local anaesthetics in cancer patients with reduced tumor metastases and recurrence [22–24], prospective, large and randomized clinical trials on the effects of regional anaesthesia on long-term outcome after cancer surgery are required. This will provide confirmation of anaesthetics’ implication in cancer patients to guide clinical practice. Our study complements the efforts to drive better understanding of its mechanisms using preclinical studies to evaluate the direct effects of local anaesthetics on representative cancer cells. Local anaesthetics can reach the circulatory system via absorption from the injection site or direct intravenous injection (eg, lidocaine) to affect circulating tumour cells released from the primary tumour during surgery. The anti-cancer activities of common amide-linked local anaesthetics have been identified in various cancers, such as lung cancer, hepatocellular carcinoma and thyroid cancer [25–27]. We previously revealed the anti-breast cancer activity of ropivacaine [20] and anti-migratory effect of bupivacaine in gastric cancer [13]. Given the fact that little is known on the effects of local anaesthetics on esophageal carcinoma cells, the present study comprehensively evaluated and compared the effects of four local anaesthetics on two well-characterized esophageal carcinoma cell lines: OE19 and SK-GT-4. We found that four local aesthetics had differential effects and mechanisms on esophageal carcinoma cell migration, growth, survival and chemosensitivity.

Metastatic disease is the most important cause of cancer-related death in patients after surgery. Using Boyden Chamber migration assay, we showed that bupivacaine and ropivacaine were more potent than lidocaine and mepivacaine in inhibiting migration (Fig. 1). The estimated IC50 of anti-migratory effect of bupivacaine, ropivacaine, lidocaine and mepivacaine in esophageal carcinoma cells are ~ 10 μM, ~ 50 μM, > 100 μM and > 100 μM. Plasma concentrations of these four local anesthetics ranged from 2.8 to 10 μM [15]. Our results suggest that bupivacaine is the only local anesthetics among the four, at clinically achievable concentration, to inhibit migration in esophageal carcinoma. In contrast, all four local anesthetics inhibited proliferation in a similar manner (Fig. 2a). Compared to anti-migratory activity, the effective concentration of anti-proliferative activity requires up to 250-time higher doses, with IC50 at ~ 2.5 mM in esophageal carcinoma cells (Fig. 2a). Although the plasma concentration of local anesthetics is at low micromolar range, local infiltration concentration of bupivacaine, ropivacaine, lidocaine and mepivacaine might reach sub-millimolar level [15]. Our results suggest that none of local anesthetics at plasma achievable concentration affects esophageal carcinoma cell proliferation. The varying effective doses of anti-migratory and anti-proliferative effects of local anesthetics observed in our study are consistent with the previous reports [28–30]. We further showed that the four local anesthetics at millimolar concentration had slight or modest pro-apoptotic effects (Fig. 2c), suggesting that local anesthetics are less likely to affect esophageal carcinoma cell survival.

Chemotherapeutic agents, such as 5-FU, cisplatin and paclitaxel, are the most frequently used chemotherapy for esophageal carcinoma [31]. Apart from the identification of local anaesthetics’ effect as single drug alone, we further revealed that local anaesthetics significantly augmented the inhibitory effects of 5-FU and paclitaxel (Fig. 3). Our results are supported by the previous work on the combinatory effects of lidocaine and bupivacaine with chemotherapy agents on cancer cells [13, 32]. This finding provides pre-clinical evidence that local anaesthetics can be considered for pain management in patient with advanced esophageal carcinoma, especially in those who are concurrently receiving chemotherapy. Of note, our work showed the differential combinatory effects of local anaesthetics with chemotherapeutic agents in different aspects of tumour cell biological functions. Additionally, two esophageal cell lines displayed differential response to the drug combination (Fig. 3). Our results also highlight that there is no common mechanism to account for the anti-esophageal carcinoma activity for all four local anaesthetics. Generally, bupivacaine and ropivacaine but not lidocaine or bupivacaine inhibited migration through decreasing activity of Rac1 (Fig. 4a-c). Rac1 exerts an important regulatory role in cell motility by formation of lamellipodia [33]. Our findings on the inhibitory effects of ropivacaine on Rac1 activity is consistent with the previous report that ropivacaine inhibits Rac1/JNK/paxillin in cancer cells [16]. Our study demonstrates the well correlated effective concentrations of local anaesthetics between proliferation and mitochondrial respiration (Fig. 2a and Fig. S2). Taken together, these suggest that local anaesthetics are likely to inhibit cell migration via inhibiting Rac1, and inhibit cell proliferation via inhibiting mitochondrial respiration. The exact molecular targets which are responsible for local anaesthetics’ differential action in esophageal carcinoma cells are worthy of further investigations.

## Conclusion

In conclusion, our findings demonstrate the direct inhibitory effects of four local anesthetics in esophageal carcinoma cells with different effective concentrations. The mechanisms of the action of local anesthetics on esophageal carcinoma cells are likely due to their ability in inhibiting Rac1, inducing mitochondrial dysfunctions, and increasing oxidative stress and damage.

## Supplementary information

**Additional file 1: Fig. S1.** Ropivacaine and bupivacaine arrest cell cycle via increasing G2/M percentage in esophageal carcinoma cells. After 24-h drug treatment, the cell cycle was assessed by staining cells with Propidium iodide (PI) and followed by flow cytometry. *P < 0.05, **P < 0.01, ***P < 0.001, compared to control. **Fig. S2.** All local anesthetics decrease OCR level in esophageal carcinoma cells. *P < 0.05, **P < 0.01, ***P < 0.001, compared to control. **Fig. S3.** All local anesthetics increase ROS level in esophageal carcinoma cells. *P < 0.05, **P < 0.01, ***P < 0.001, compared to control. **Fig. S4.** All local anesthetics increase 8-OHdG level in esophageal carcinoma cells. *P < 0.05, **P < 0.01, ***P < 0.001, compared to control.

## Data Availability

The datasets used and/or analysed during the current study available from the corresponding author on reasonable request.

## Abbreviations

JNK: c-Jun N-terminal kinase
FBS: Fetal bovine serum
STR: Short tandem repeat
5-FU: 5-Fluorouracil
ANOVA: One-way analysis of variance
8-OHdG: 8-hydroxydeoxyguanosine
OCR: Oxygen consumption rate
