# Supplementary material for: Differential effects and mechanisms of local anesthetics on esophageal carcinoma cell migration, growth, survival and chemosensitivity
Source: BMC Anesthesiol. 2020 May 25;20:126. doi: 10.1186/s12871-020-01039-1 (PMC7249391; doi:10.1186/s12871-020-01039-1)
Supplement: Supplementary file 1 — Additional file 1: Fig. S1. Ropivacaine and bupivacaine arrest cell cycle via increasing G2/M percentage in esophageal carcinoma cells. After 24-h drug treatment, the cell cycle was assessed by staining cells with Propidium iodide (PI) and followed by flow cytometry. *P < 0.05, **P < 0.01, ***P < 0.001, compared to control. Fig. S2. All local anesthetics decrease OCR level in esophageal carcinoma cells. *P < 0.05, **P < 0.01, ***P < 0.001, compared to control. Fig. S3. All local anesthetics increase ROS level in esophageal carcinoma cells. *P < 0.05, **P < 0.01, ***P < 0.001, compared to control. Fig. S4. All local anesthetics increase 8-OHdG level in esophageal carcinoma cells. *P < 0.05, **P < 0.01, ***P < 0.001, compared to control. [file 12871_2020_1039_MOESM1_ESM.doc]

**Differential effects and mechanisms of local anesthetics on esophageal carcinoma cell migration, growth, survival and chemosensitivity**

**Fig. S1: Ropivacaine and bupivacaine arrest cell cycle via increasing G2/M percentage in esophageal carcinoma cells.** After 24-hour drug treatment, the cell cycle was assessed by staining cells with Propidium iodide (PI) and followed by flow cytometry. *P< 0.05, **P< 0.01, ***P< 0.001, compared to control.

**Fig. S2: All local anesthetics decrease OCR level in esophageal carcinoma cells.** *P< 0.05, **P< 0.01, ***P< 0.001, compared to control.

**Fig. S3: All local anesthetics increase ROS level in esophageal carcinoma cells.** *P< 0.05, **P< 0.01, ***P< 0.001, compared to control.

**Fig. S4: All local anesthetics increase 8-OHdG level in esophageal carcinoma cells.** *P< 0.05, **P< 0.01, ***P< 0.001, compared to control.
